# Functional outcomes after total joint arthroplasty are related to the severity of Parkinson’s disease: a mid-term follow-up

**DOI:** 10.1186/s13018-019-1447-8

**Published:** 2019-11-28

**Authors:** Xiao Rong, Suraj Dahal, Ze-yu Luo, Kai Zhou, Shun-Yu Yao, Zong-Ke Zhou

**Affiliations:** Department of Orthopedics, West China Hospital/West China School of Medicine, Sichuan University, Chengdu, 610041 People’s Republic of China

**Keywords:** Parkinson’s disease, Total joint arthroplasty, Functional outcomes

## Abstract

**Background:**

Performing total joint arthroplasty (TJA) in Parkinson’s disease (PD) patients may encounter a higher complication rate or worse functional outcomes compared with common patients. The relationship between PD and clinical outcomes after TJA is not fully understood.

**Methods:**

Retrospectively**,** we used manual charts to investigate the clinical outcomes in 41 patients including 24 total hip arthroplasty (THA) patients (28 hips) and 18 total knee arthroplasty (TKA) patients (22 knees) with a diagnosis of PD from 2009 to 2016. The stage of PD was confirmed by Hoehn and Yahr scale. Prosthesis survivorship was estimated with revision for any reason as the endpoint.

**Result:**

All the clinical outcomes improved significantly (*p* < 0.05). Subgroup analysis revealed worse functional outcomes in mid- or end-stage PD patients. Sixteen short-term mild to moderate complications were noted. Two revisions were conducted for hip periprosthetic osteolysis and postoperative knee pain. The prosthesis survivorship at 60 months for TJA, total hip arthroplasty (THA), or total knee arthroplasty (TKA) was 91.6%, 94.1%, and 87.5%, respectively.

**Conclusion:**

Patients with PD who underwent TJA would result in excellent pain relief and gain of function. However, patients at late-stage PD may suffer from functional loss. The effectiveness of TJA in patients with severe PD remains a concern. Physician should help delay the progression of PD which may optimize and stabilize the functional outcomes of TJA.

## Introduction

Parkinson’s disease (PD) is the second most common neurodegenerative disease (after Alzheimer’s disease) with a life expectancy of 7–14 years [1]. The incidence of PD in high-income countries is 14 per 100,000 and 160 per 100,000 in people aged over 65 years [2]. The motor symptoms of PD are the most prominent including bradykinesia, muscular rigidity, rest tremor, and postural and gait impairment [1]. Many PD patients demand lower limb total joint arthroplasty (TJA) for different reasons including end-stage osteoarthritis (OA), fracture, or osteonecrosis of the femoral head [3]. With the motor symptoms, the surgical outcomes may be affected.

The connection between PD and the potential requirement of TJA has been studied. Gait abnormality and instability mean a higher rate of fall of patients with PD. The study suggested that 60.5% of participants reported at least one fall, with 39% reporting recurrent falls [4]. A relevant study confirmed 4.48 times more likely of PD patients experienced hip fracture caused by severe falls [5, 6] which may sustain primary total hip arthroplasty or revision surgeries. Prior research showed that patients with PD and OA seemed suffered from akathisia, paraesthesia, and heightened pain level in comparison with OA alone [7]. Besides, previous researches have demonstrated a higher rate of complications after THA or TKA could be seen in patients with PD.

PD is a progressive disease characterized by worsening of motor features [1]. Hoehn and Yahr (H-Y) scale (Table 1) is the most commonly used form to define the stage of PD since 1967 [8]. The stage progresses with a median time range from 20 to 62 months while the motor symptoms worsen with progression [9]. At the end stage of PD (H-Y stage 5), usually with a course of 10–20 years, patients would be bed or wheelchair bounded and require home nursing [10, 11]. Therefore, the outcomes of TJA may be affected by severe functional loss.

**Table 1:** Hoehn and Yahr Scale for Parkinson’s disease

Stage	Hoehn and Yahr Scale
I	Unilateral involvement only
II	Bilateral involvement without impairment of balance
III	Mild to moderate bilateral disease; some postural instability; physically independent
IV	Severe disability; still able to walk or stand unassisted
V	Wheelchair bound or bedridden unless aided

Therefore, this study aims to demonstrate the mid-term pain and functional outcomes of PD patients who underwent THA or TKA. Further subgroup analysis would be done to demonstrate the potential relationship between the stage of PD and the outcomes of patients.

## Materials and methods

### Patients selection

After the approval of the Institutional Review Board of West China Hospital, this retrospective study was conducted at West China Hospital, Sichuan University, which serves as a tertiary level center and the largest medical center in the southwest China. Patients with the diagnosis of PD who underwent primary total hip or knee arthroplasty between June 2009 and June 2016 were included in our study and established TKA and THA groups afterward. The informed consent was obtained. Patients who meet the following conditions would be excluded: (1) not primary joint arthroplasty, (2) patient with other neurological diseases, and (3) patient with severe or uncontrolled comorbidities.

A total of 56 consecutive patients were primarily included in our study. Among them, seven patients were failed to follow-up due to the incorrect telephone numbers, four patients were excluded for incomplete chart filling, and four patients were excluded for not being primarily admitted. No demise happened during follow-up. Therefore, 41 patients including 24 THA patients (28 hips) and 18 TKA patients (22 knees) were finally enrolled in the study. One of the patients received both THA and TKA successively. The median duration of follow-up is 41.00 months (IQR 32.00–67.75) for all the candidates and, separately, 51.50 months (IQR 34.00–72.00) for THA patients and 38.00 months (IQR 29.75–50.75) for TKA patients. There were 14 males and 31 females with an average age of 67.86 years (range, 47–85). The body mass index (BMI) was 22.73 kg/m^2^ in average (range, 15.63–31.11). More baseline demographics could be referred to in Table 2.

**Table 2:** Baseline characteristics and Hoehn and Yahr stage

Variables	THA group	TKA group
Number of patients	24	18
Number of hips	28	22
Gender		
Male	8 (33.33%)	3 (16.67%)
Female	16 (66.67%)	15 (83.33%)
Weight (kg), mean ± SD	54.73 ± 10.12	59.09 ± 9.96
Height (cm), mean ± SD	158.04 ± 6.89	157.28 ± 7.43
BMI, mean ± SD	21.91 ± 3.82	23.83 ± 3.25
Age, mean (range)	64.67 ± 10.69 (47–80)	67.89 ± 6.62 (56–85)
Follow-up (months), median (IQR)	51.50 (34.00–72.00)	38.00 (29.75–50.75)
Side		
Left	15 (53.57%)	11 (50.00%)
Right	13 (46.43%)	11 (50.00%)
Hoehn and Yahr stage		
I	7 (29.17%)	2 (11.11%)
II	5 (20.83%)	7 (38.89%)
III	8 (33.33%)	5 (27.78%)
IV	3 (12.50%)	4 (22.22%)
V	1 (4.15%)	0

*Abbreviations*: *THA* total hip arthroplasty, *TKA* total knee arthroplasty, *SD* standard deviation, *BMI* body mass index, *IQR* interquartile range

### Clinical data

The collection of the clinical data and evaluation was conducted by two independent observers (X-R and ZY-L). Our patients would undergo routine follow-up at postoperatively 3 weeks, 6 weeks, 12 weeks, and 6 months and annually. And manual chart interview was adopted for evaluation of the preoperative and postoperative clinical outcomes. Western Ontario and McMaster Universities Osteoarthritis Index (WOMAC) (pain, stiffness, and function) and The Short Form (12) Health Survey scale (SF-12) (physical component summary (PCS) and mental component summary (MCS)) were used for all the candidates. Respectively, for THA patients, Harris Hip Score (HHS) was used to evaluate the pain, function, and deformity. Moreover, for TKA patients, Knee Society Score (KSS) and Knee Society Function Score (KSFS) were used in terms of stability, pain, deformity, and function evaluation. The multiple scales provided a more comprehensive assessment which would cover pain and function, mental and physical state, daily activity, quality of life, and symptoms. Other evaluation terms including the preoperative and postoperative range of motion (ROM) and the severity of PD was assessed by Hoehn and Yahr scale [8] (Table 1).

### Subgroup analysis

PD patients at H-Y stage I or II could keep balance, remain independent, and be functional free. However, at stage III, patients would develop postural instability with mild functional restriction. At stage IV or V, these symptoms deteriorate, and severe disability could be seen. Based on this phenomenon, we enrolled patients at stage I or II into the functional free group (group I). Patients at stage III to V were enrolled in the functional restriction group (group II). The clinical outcomes were compared between them.

### Statistical analysis

Quantitative data were presented as means and standard deviations or as median with the interquartile ranges (IQR 25th and 75th percentiles). Qualitative data were presented with frequencies and percentages. Student’s *t* test was used to compare the continuous variables while Pearson chi-square test or Fisher exact test was used to analyzing the qualitative comparative variables. For the prosthesis survival analysis, Kaplan-Meier was utilized with any reason as the endpoints for revisions. Spearman correlation analysis was adopted for demonstrating the relationship between ranked data and continuous data. The significance was set at *p* < 0.05. We used SPSS for Windows (Version 21.0 IBM Corp, Armonk, NY, USA) to perform all the statistical analyses.

## Result

### Clinical outcomes

The outcomes with regard to hip or knee pain and functions were listed in Table 3. For patients who underwent THA, Harris Hip Score (HHS) demonstrated a significant improvement. The total score improved from 39.00 ± 15.74 preoperatively to 71.39 ± 19.18 postoperatively (*p* < 0.01). Moreover, in specific terms of pain (18.75 ± 9.92 vs. 38.83 ± 9.26, *p* < 0.01), function (18.08 ± 7.17 vs. 28.83 ± 13.10, *p* < 0.01), and deformity (2.17 ± 0.92 vs. 3.73 ± 0.44, *p* < 0.01), the improvement was obvious as well.

**Table 3:** Clinical outcomes from patients

Variable mean ± SD	THA group, *N* = 24		*P*	TKA group, *N* = 18		*P*
	Preoperative	Postoperative		Preoperative	Postoperative	
HHS						
Pain	18.75 ± 9.92	38.83 ± 9.26	< 0.01	–	–	–
Function	18.08 ± 7.17	28.83 ± 13.10	< 0.01	–	–	–
Deformity	2.17 ± 0.92	3.73 ± 0.44	< 0.01	–	–	–
Total	39.00 ± 15.74	71.39 ± 19.18	< 0.01	–	–	–
KSS						
Pain	–	–	–	21.11 ± 9.63	43.61 ± 12.10	< 0.01
ROM	–	–	–	22.22 ± 1.86	23.61 ± 1.61	< 0.01
Stability	–	–	–	17.89 ± 2.00	24.11 ± 1.18	< 0.01
Total	–	–	–	61.22 ± 9.66	91.33 ± 12.57	< 0.01
KSFS	–	–	–	39.72 ± 6.52	58.06 ± 31.11	0.02
SF-12						
PCS	13.96 ± 2.54	17.54 ± 6.09	0.01	15.61 ± 3.55	20.17 ± 5.08	< 0.01
MCS	17.96 ± 2.82	21.41 ± 6.25	0.02	20.28 ± 3.49	24.00 ± 5.16	0.02
WOMAC						
Pain	10.63 ± 3.51	2.96 ± 4.13	< 0.01	8.39 ± 2.55	3.39 ± 4.55	< 0.01
Stiffness	4.25 ± 1.85	1.75 ± 1.85	< 0.01	2.94 ± 1.30	0.61 ± 1.50	< 0.01
Function	44.08 ± 7.90	34.63 ± 15.52	< 0.01	38.00 ± 6.73	26.44 ± 15.24	< 0.01
ROM						
Flexion	70.42 ± 40.59	111.13 ± 15.16	< 0.01	111.11 ± 9.32	118.33 ± 8.22	< 0.01
Abduction	21.88 ± 16.93	38.29 ± 6.21	< 0.01	–	–	–

*Abbreviation*s: *SD* standard deviation, *THA* Total hip arthroplasty, *TKA* total knee arthroplasty, *HHS* Harris Hip Score, *KSS* Knee Society Score, *KSFS* Knee Society Function Score, *SF-12* Short Form (12) Health Survey scale, *PCS* physical component summary, *MCS* mental component summary, *WOMAC* Western Ontario and McMaster Universities Osteoarthritis Index, *ROM* range of motion

In the aspect of TKA, Knee Society Score showed the same trend of elevation from 61.22 ± 9.66 preoperatively to 91.33 ± 12.57 postoperatively (*p* < 0.01). Pain (21.07 ± 8.32 vs. 43.61 ± 12.10, *p* < 0.01) and stability (17.89 ± 2.00 vs. 24.11 ± 1.18, *p* < 0.01) showed a significant rise while the ROM score (22.22 ± 1.86 vs. 23.61 ± 1.61, *p* < 0.01) presented a slit elevation but is also statistically significant. KSFS revealed functional relief from 39.72 ± 6.52 to 58.06 ± 31.11 (*p* = 0.02). Further, WOMAC, SF-12, and ROM were reviewed, and all showed statistically significant improvement (Table 3).

### Short-term complications

Six hips (28.43%) and six knees (27.27%) showed 16 mild short-term complications (Table 4). One of the hips (3.57%) experienced intraoperative femoral fracture and were cured with cerclage cable. Upper respiratory infection occurred in two hip cases (7.14%) and one knee case (4.55%) and was cured with antivirus, antibiotic, or atomization inhalation. Two knee cases (9.09%) presented nausea or sour regurgitation while one hip case (3.57%) complained of vomiting. They were controlled with gastroprokinetic agents or omeprazole. Bruise happened in two hips (7.14%) and three knees (13.64%). All of them recovered after withdrawal or reducing the dosage of anticoagulant. One knee (4.55%) presented lower limb swelling which was handled with functional training and rehabilitation. Other short-term complications include delirium (one hip 3.57% and one knee 4.55%), which was controlled with olanzapine, and mild anemia (1 hip, 3.53%) with proper iron agent treatment. No evidence of embolism, hematoma, or nerve palsy was observed.

**Table 4:** Complications

Complications	THA group	TKA group
Intraoperative fracture	1 (3.57%)	0
Gastrointestinal disorder	1 (3.57%)	2 (9.09%)
Lower limb swelling	0	1 (4.55%)
Bruise	2 (7.14%)	3 (13.64%)
Upper respiratory infection	2 (7.14%)	1 (4.55%)
Delirium	1 (3.57%)	1 (4.55%)
Anemia	1 (3.57%)	0
Embolism	0	0
Hematoma	0	0
Nerve palsy	0	0

*Abbreviation*s: *THA* total hip arthroplasty, *TKA* total knee arthroplasty

### Survivorship analysis

One patient suffered from knee pain after the TKA and was cured with humeral replacement at 44 months postoperatively. Another patient in the THA group presented periprosthetic osteolysis and polyethylene wear at 42 months after primary THA and was treated with revision surgery. No evidence of periprosthetic infection, dislocation, aseptic loosening, or other complications occurred during the follow-up.

**Fig. 1:**
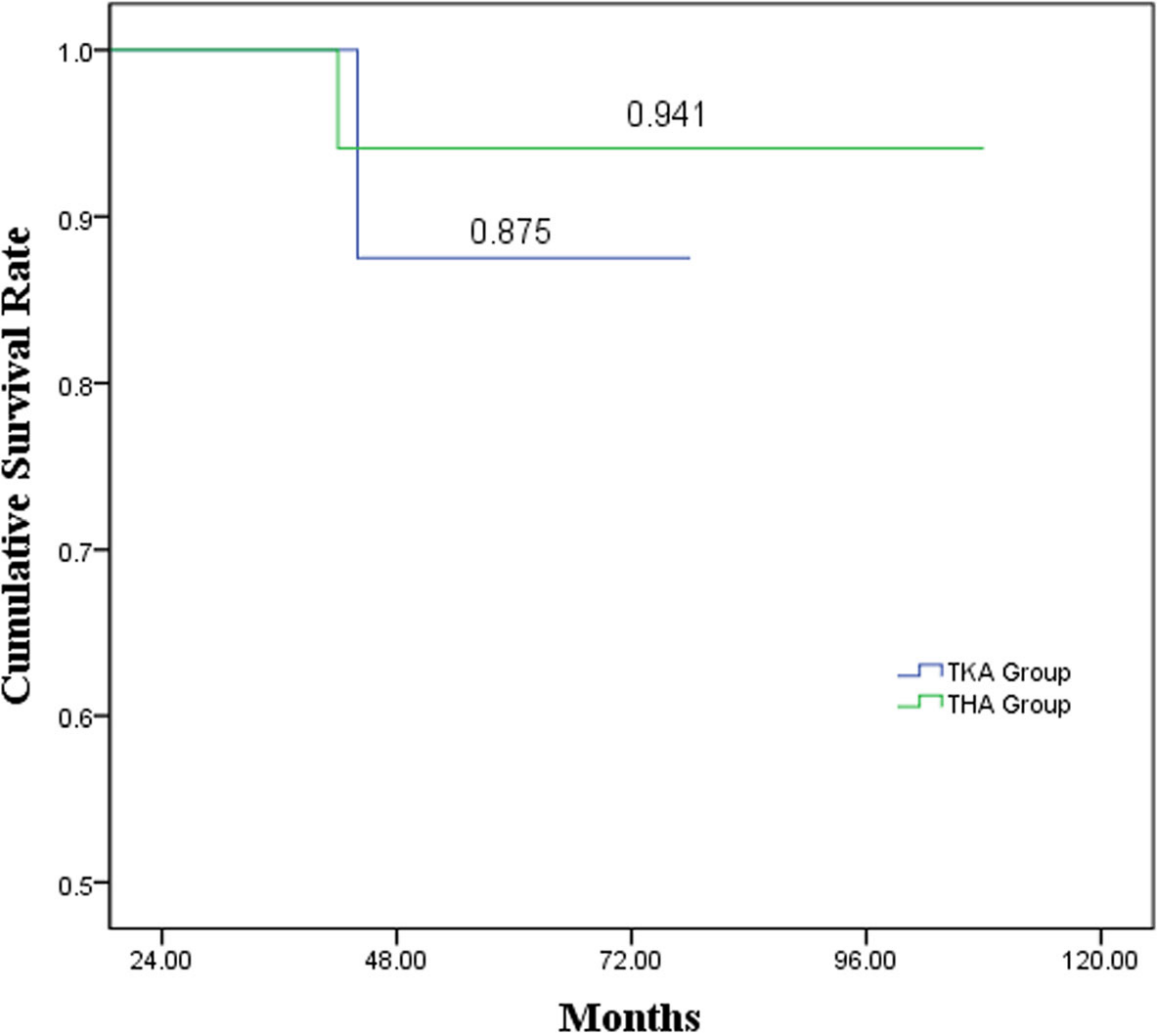
A Kaplan-Meier survivorship cure for THA and TKA group

Using revision for any reason as the end point, the overall survivorship rate of the lower limb arthroplasty at 60 months was 91.6% (95% CI 80.4–99.9%). Also, for the THA group and TKA group respectively, the 60 months survivorship rate was 94.1% (95% CI 82.9–99.9%) and 87.5% (95% CI 64.6–99.9%) (Fig. 1).

### Subgroup analysis

The SF-12 score (PCS and MCS *p* < 0.01) and the WOMAC pain (*p* = 0.01) and function score (*p* < 0.01) revealed significant improvement in group II for the entire case series. Respectively, in the THA group, the HHS score (*p* = 0.03) and function score (*p* = 0.03), SF-12 score (PCS *p* = 0.02, MCS *p* < 0.01), and the WOMAC function score (*p* < 0.01) were statistically different between subgroups (Table 5). Of the patients in TKA group, the KSFS (*p* < 0.01), SF-12 (PCS *P* < 0.01, MCS *p* < 0.01), and the WOMAC pain (*p* = 0.03) and function score (*p* < 0.01) were significantly higher in group II. Furthermore, comparisons of the final clinical data with preoperative data were done in each group. Significant differences of all the data were seen in group I. However, in group II, there were no differences in HHS function score (*p* = 0.25) and KSFS (*p* = 0.50). The SF-12 (THA: PCS, *p* = 0.69 and MCS, *p* = 0.61; TKA: PCS, *p* = 0.10 and MCS, *p* = 0.26) and WOMAC function score (THA: *p* = 0.93; TKA: *p* = 0.74) were not statistically different as well.

**Table 5:** Subgroup clinical outcomes comparison

Variables mean ± SD	THA		*P*	TKA		*P*
	Group I, *N* = 12	Group II, *N* = 12		Group I, *N* = 9	Group II, *N* = 9	
HHS						
Total	79.58 ± 13.94	63.21 ± 20.71	0.03	–	–	–
Pain	41.5 ± 4.10	36.17 ± 12.13	0.16	–	–	–
Deformity	3.75 ± 0.45	3.71 ± 0.45	0.82	–	–	–
Function	34.33 ± 11.42	23.33 ± 12.74	0.03	–	–	–
KSS						
Pain	–	–	–	47.78 ± 3.63	39.44 ± 16.09	0.15
ROM	–	–	–	24.33 ± 0.71	22.89 ± 1.96	0.05
Stability	–	–	–	24.56 ± 0.88	23.67 ± 1.32	0.11
Total	–	–	–	96.78 ± 3.53	86.00 ± 16.12	0.07
KSFS	–	–	–	81.11 ± 14.53	35.00 ± 25.50	< 0.01
SF-12						
PCS	20.42 ± 5.21	14.67 ± 5.69	0.02	22.78 ± 3.07	17.56 ± 5.48	0.02
MCS	25.92 ± 3.99	16.92 ± 4.64	< 0.01	27.00 ± 2.06	21.00 ± 5.66	0.01
WOMAC						
Pain	1.83 ± 2.72	4.08 ± 5.05	0.19	1.11 ± 1.27	5.67 ± 5.55	0.03
Stiffness	1.33 ± 1.72	2.17 ± 1.95	0.28	0.33 ± 0.71	1.00 ± 2.00	0.36
Function	25.83 ± 12.88	43.42 ± 13.01	< 0.01	16.22 ± 9.96	36.67 ± 12.61	< 0.01
ROM						
Flexion	115.91 ± 12.13	106.33 ± 16.82	0.12	24.33 ± 0.71	22.89 ± 1.96	0.05
Abduction	40.42 ± 5.42	36.17 ± 6.43	0.09	N/A	N/A	N/A

*Abbreviations*: *SD* standard deviation, *THA* total hip arthroplasty, *TKA* total knee arthroplasty, *HHS* Harris Hip Score, *KSS* Knee Society Score, *KSFS* Knee Society Function Score, *SF-12* Short Form (12) Health Survey scale, *PCS* physical component summary, *MCS* mental component summary, *WOMAC* Western Ontario and McMaster Universities Osteoarthritis Index, *ROM* range of motion.

**Fig. 2:**
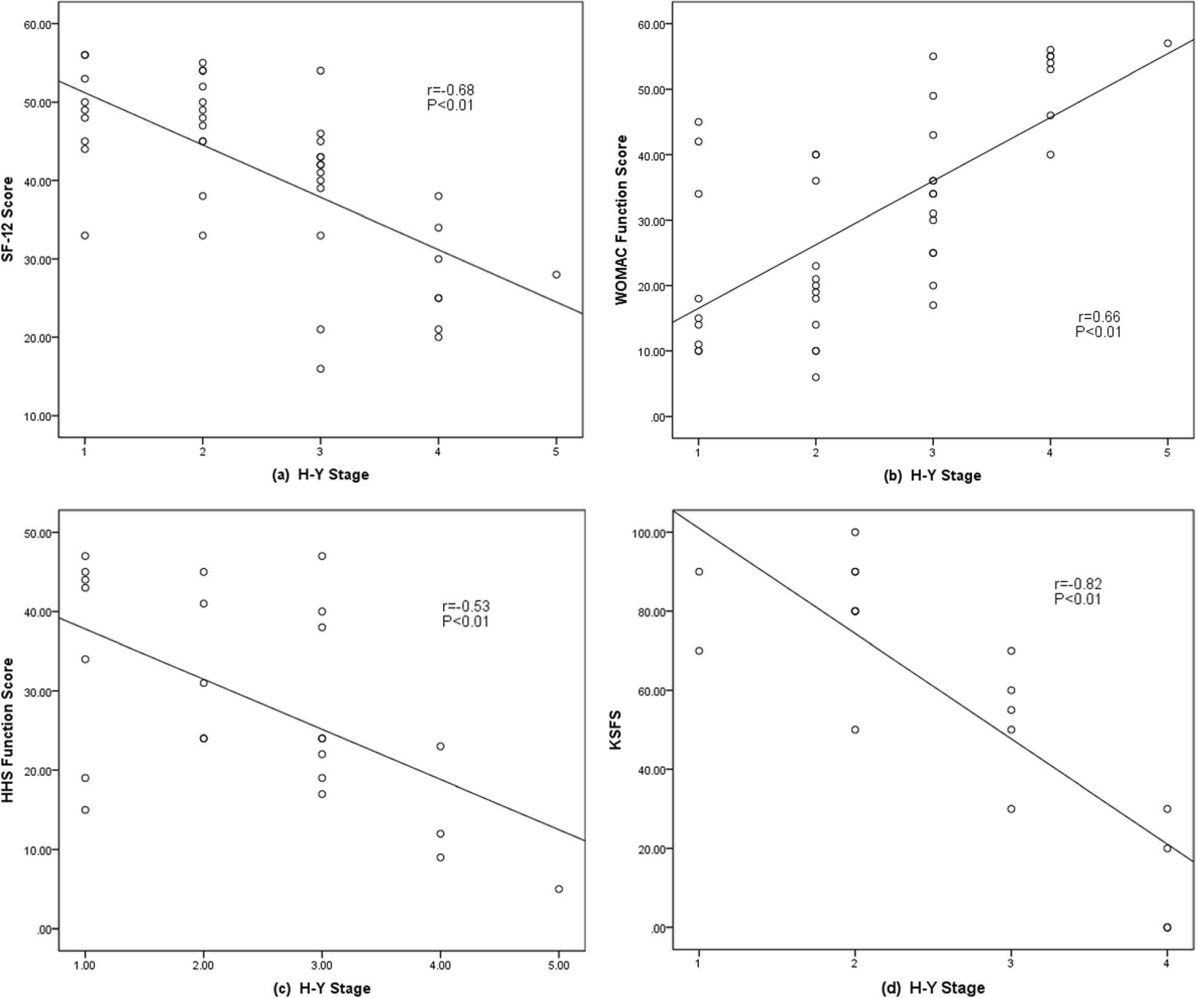
Scatter diagram and the fitted lines of functional outcomes and H-Y stages. **a** Correlation between SF-12 score and H-Y stages. **b** Correlation between WOMAC Function score and H-Y stages. **c** Correlation between HHS Function score of THA patients and H-Y stages. **d** Correlation between KSFS of TKA patients and H-Y stages

Spearman correlation analysis was done to demonstrate the relationship between functional outcomes and the H-Y stage of the patient. For all the candidates, the SF-12 score (*r* = − 0.67, *p* < 0.01) and WOMAC function score (*r* = 0.64, *p* < 0.01) correlated with the H-Y stage. The HHS function score (*r* = − 0.53, *p* < 0.01) in the THA group and KSFS (*r* = − 0.82, *p* < 0.01) in the TKA group also demonstrate the same result. The scatter diagrams and the fitted lines are presented in Fig. 2.

## Discussion

Total joint arthroplasty in patients with Parkinson’s disease (PD) could be a challenge in functional regaining due to the high risk of fall [4], bone fractures [5, 6], and an increased incidence of osteoporosis [12, 13]. In this study, we retrospectively investigated the clinical outcomes in patients with PD who underwent TKA or THA. We showed both pain and functional outcomes improved significantly after a median follow-up of 41.00 months (IQR 32.00–67.75). We also demonstrated the functional outcomes of PD patients were related to the severity of PD. The result suggested PD patients at advanced stages result in poor function scores compared with early stages. In addition, the functional outcomes of the late-stage patients revealed no significant difference when compared with preoperative data. To explain this phenomenon, we considered the reasons as follows. Firstly, the establishment of the subgroups was based on functional performance. The onset of functional restriction starts at stage III and deteriorate with motor symptoms. Secondly, late-stage non-motor symptoms including depression and sleep disorders worsen the mental state of the patients [14, 15]. Finally, the scales are consist of walking distance, aids usage, stairs climbing, and other daily activities that are highly affected by the motor symptoms. Moreover, the SF-12 scales include the mental health assessment which is affected by the non-motor symptoms. Previous studies presented conflict conclusions with regard to functional outcomes. One study presented improvement in SF-12 scores in PD patients who underwent TJA, but it was greater in the control cohort [16]. Certain studies confirmed poor functional improvement in PD patients after TJA [17, 18], and two studies indicate the functional results depend on the severity and progression of PD [18, 19].

We find mild to moderate complications with acceptable rate happened in this case series. There was no evidence of DVT, embolism, or nerve palsy. Two of our patients suffered from transient delirium and were controlled with olanzapine, which confirmed that orthopedic surgery could induce a cognitive decline in PD patients [20]. Recently, some database studies [21–26] showed both TKA and THA in PD patients provide a higher complication rate, longer LOS, and higher charges than the controlled cohort. One study [27] considered the higher complication rate of PD patients was associated with cardiovascular and psychiatric comorbidity. Furthermore, we found excellent prosthetic survivorship in PD patients (60 months, 91.6% (95% CI 80.4–99.9%)). However, previous studies [16, 27] suggested TJA in PD patients resulted in a higher risk of revision or hip dislocation. Recurrent posterior dislocation was also reported in PD patients [28].

Lower limb muscle weakness is common in both postoperative TJA [29, 30] and PD patients [31]. As the adoption of physical exercise and training in improving limb function after TJA, a rehabilitation program is also suitable for patients with PD. A systematic review has confirmed that progressive resistance exercise could effectively improve the quality of daily activities of PD patients [32]. Meanwhile, power-based resistance training could significantly reduce bradykinesia and increased muscle strength and power in older patients with PD [33]. Based on our findings, the stage of the PD patients and the motor symptoms are highly connected with the functional outcomes of the TJA. Future studies may focus on the rehabilitation programs in relieving motor symptoms, delaying stage progression, and thus improving the function of the PD patients.

There are certain limitations to this study. First, the outcomes of our patients were based on conventional questionnaires. It would be more objective and reliable if a quantitative gait analysis were conducted for the patients. Second, we investigated both hip and knee prosthesis in one study. However, the evaluation forms were used correspondingly and all the data were presented in separate groups.

## Conclusion

Patients with PD who underwent TJA would result in excellent pain relief and gain of function. However, patients at late-stage PD may suffer from functional loss. The effectiveness of TJA in patients with severe PD remains concern. Physician should help delay the progression of PD which may optimize and stabilize the functional outcomes of TJA.

## Data Availability

The datasets used and analyzed during the current study are available from the corresponding author on reasonable request.
